# Organizational readiness and implementation of colorectal cancer screening evidence-based interventions in federally qualified health centers: A cross-sectional study

**DOI:** 10.1017/cts.2024.689

**Published:** 2024-12-26

**Authors:** Emanuelle M. Dias, Timothy J. Walker, Bijal A. Balasubramanian, Paula M. Cuccaro, Lauren Workman, Abraham Wandersman, Maria E. Fernandez

**Affiliations:** 1 UTHealth Houston School of Public Health, Houston, TX, USA; 2 UTHealth Houston Institute for Implementation Science, UTHealth Houston School of Public Health, Houston, TX, USA; 3 Center for Applied Research and Evaluation, Arnold School of Public Health, University of South Carolina, Columbia, SC, USA; 4 Department of Health Services, Policy, and Management, Arnold School of Public Health, University of South Carolina, Columbia, SC, USA; 5 Wandersman Center, Columbia, SC, USA

**Keywords:** Organizational readiness, implementation, colorectal cancer screening, evidence-based interventions, federally qualified health centers, organizational structure

## Abstract

**Introduction:**

Evidence-based interventions (EBIs) exist to increase colorectal cancer (CRC) screening, but implementation remains slow in federally qualified health centers (FQHCs). Assessing organizational readiness can improve EBI implementation outcomes, but no studies have quantitatively examined the relation between organizational readiness subcomponents and implementation outcomes. This study examines associations between readiness subcomponents and CRC screening EBI implementation outcomes in FQHCs.

**Methods:**

We used data from an ongoing parent study to develop an organizational readiness measure using the *R* = MC^2^ heuristic. We conducted descriptive and cross-sectional analyses using FQHC clinic (*n* = 57) data across three states. A clinic contact completed a survey about clinic characteristics and then distributed an EBI-specific survey to clinic staff containing readiness and implementation questions about *Community Guide* EBIs (e.g., patient reminders). Pearson correlations assessed bivariate associations between readiness variables and implementation outcomes. We then computed multivariable linear associations between readiness variables and implementation outcomes while controlling for clinic-level variables. One-way analysis of variance tested group differences in readiness subcomponent mean scores using EBI implementation responses.

**Results:**

Respondents’ most common job type was medical assistant, and the most frequently implemented EBIs were provider or patient reminders. Organizational structure was associated with implementing patient reminders. Clinics reporting inconsistent implementation had lower organizational structure scores than clinics planning or fully implementing patient reminders.

**Conclusion:**

This study guides researchers in prioritizing organizational structure and selecting specific implementation strategies to improve this construct to implement CRC screening-related EBIs. Future research should examine these associations using a larger sample size to explore additional relations between organizational readiness and implementation outcomes.

## Introduction

The Guide to Community Preventive Services (i.e., *The Community Guide*) is a systematic resource of empirically derived evidence-based interventions (EBIs) for increasing colorectal cancer (CRC) screening. These include patient and provider reminders, reducing structural barriers (e.g., addressing transportation or language translation barriers), and provider assessment and feedback, among others [1]. These EBIs are also supported by the Centers for Disease Control and Prevention (CDC)’s Colorectal Cancer Control Program, aimed at increasing screening among individuals aged 45 to 75 years. Although EBIs can potentially increase CRC screening across settings, their adoption and implementation remain slow and inconsistent in federally qualified health centers (FQHCs) compared to other clinical settings [2,3].

Previous studies have uncovered some determinants of implementing CRC screening EBIs in FQHCs, including limited resources, technology, sustainability issues, external partnerships, communication challenges, competing priorities, and staff time [4–7]. Therefore, there is a pressing need to improve the adoption and implementation of EBIs for CRC screening in FQHCs. To increase implementation, it is essential to first understand organization-level factors potentially influencing the uptake and use of EBIs for CRC screening. Understanding organization-level factors, also known as *determinants* or *barriers* and *facilitators* of implementation, can help guide efforts to improve the implementation of CRC screening EBIs in FQHCs. *Organizational readiness* is a determinant framework[8,9] that can guide the analysis of barriers and facilitators in improving the implementation of EBIs in FQHCs.

*Organizational readiness* is an essential aspect of successful implementation across settings [9]. The organizational readiness heuristic, denoted by *R* = MC^2^ (**R**eadiness = **M**otivation × Innovation-Specific ***C***apacity × General ***C***apacity), posits that three components make up an organization’s “readiness” to implement an innovation, policy, program, or practice [9]. *Motivation* refers to the willingness of an organization to implement an innovation, *innovation-specific capacity* represents the specific skills and resources needed to implement a particular innovation, and *general capacity* describes an organization’s overall functioning to support implementation [9]. The three readiness components comprise several subcomponents (organization-level factors) that can facilitate or impede implementation. Implementation outcomes refer to the effects (how well or how much) of actions taken to implement innovations [10,11]. Evidence suggests that assessing and building organizational readiness can improve the implementation outcomes of EBIs, which is critical for facilitating implementation efforts [12].

In previous studies on understanding organizational readiness-related barriers and facilitators, the following *R* = MC^2^ subcomponents were highly relevant to implementing practice changes related to CRC screening in FQHCs: *priority* (importance of an innovation compared to other things the organization is doing), *compatibility* (how well an innovation aligns with existing needs), *observability* (seeing an innovation’s visible and desired outcomes), inter- and intra-organizational relationships (the necessary relationships within and between organizations), organizational structure (organizational communication and processes), and resource utilization (the ability to acquire and allocate resources) [12–17]. However, no studies have quantitatively examined the relation between organizational readiness subcomponents and reported levels of implementation in the clinical context. This study aimed to examine the associations between readiness subcomponents and reported levels of implementation of CRC screening EBIs in FQHCs.

## Materials and methods

### Parent study

The data used for the present study are from an ongoing parent study that aims to develop an organizational readiness measure based on the *R* = MC^2^ heuristic, which will be applicable across settings. Data collection methods and other details of the parent study have been previously published [8]. The study sample for this analysis came from the developmental phase of the parent study for which the research team distributed an expanded version of the readiness measure (including all questions in the item pool for each subcomponent) to a sample of FQHC clinics and staff across the U.S. Data for the parent study were collected using two surveys distributed via REDCap to FQHC clinics.

The team recruited participants using a convenience sampling approach, identified an FQHC contact, and asked them to complete a *clinic contact survey.* The *clinic contact survey* asked about the number of clinic sites and personnel in the FQHC system, screening rates, CRC screening tests used, EBI use in the clinic, and the extent/level of EBI implementation. After the clinic contact completed the survey, the team examined the survey and asked the clinic contact to distribute an EBI-specific survey to other clinic members.

Based on the priorities articulated in the CDC Colorectal Cancer Control Program, we asked about four EBIs – provider reminders, patient reminders, provider assessment and feedback, and reducing structural barriers – in the surveys. If the clinic contact reported that the clinic was using more than one EBI, we randomly assigned them to one of the EBI-specific surveys. For example, if clinic A used both patient and provider reminders, we used a random number generator to select one of the two readiness surveys to send to the clinic contact, who then distributed the survey link via email to employees (e.g., providers, nurses, clinic managers) directly involved in the CRC screening process across the clinic. Data were collected in three U.S. states (Texas, South Carolina, and New York) from August 2021 to April 2022. The Committee for the Protection of Human Subjects at the UTHealth Houston School of Public Health approved all the study procedures and protocols (HSC-SPH-18-0006).

### Present study

For the present study, we used cross-sectional data from the clinic contact and EBI-specific surveys to examine whether organizational readiness subcomponent scores relate to reported levels of CRC screening EBI implementation. To denote the extent of EBI implementation, we used the following item from the clinic contact survey: “Does this clinic use (e.g., provider reminders) to promote colorectal cancer screening?” with the following response categories: 1) No, and we have no plans to do so, 2) No, but we are planning to implement this strategy in the future, 3) Yes, we are at an early stage of implementing this strategy across the clinic, 4) Yes, we have implemented this strategy, but it is inconsistently implemented across the clinic, and 5) Yes, we have implemented this strategy fully and systematically across the clinic. If clinics answered that they were at least *planning to implement the strategy in the future,* this indicated that the clinic would be eligible to respond to the remaining questions related to the EBI. Below, we describe the specific variables from the parent survey used in this study.

## Measures

### Demographic questions

All survey participants answered demographic questions as part of their readiness surveys (e.g., clinic role, level of education). In addition, to gauge participant eligibility, we asked: “With regard to the implementation of colorectal cancer screening strategies at your clinic, how confident are you in your ability to answer questions about (e.g., provider reminders)?” Response options were: 1) not at all confident, 2) slightly confident, 3) somewhat confident, and 4) very confident; individuals who selected “not at all confident” were deemed ineligible to participate in the readiness survey.

### Reported levels of implementation (dependent/outcome variable)

As part of the readiness survey, we asked one question to represent the reported level of implementation of each respective EBI (provider or patient reminders, provider assessment and feedback, and reducing structural barriers). For example, we asked, “Does your clinic use *patient reminders* to promote colorectal cancer screening?” and “Does this clinic use *approaches that reduce structural barriers* to promote colorectal cancer screening?” By treating this variable as continuous, we calculated the average clinic-level score for each EBI based on the reported level of the implementation scale.

### Readiness subcomponent survey questions (independent/exposure variable)

For the present study, we utilized a selected set of seven out of a total of 18 readiness variables: priority, compatibility, observability, intra-organizational relationships, inter-organizational relationships, organizational structure, and resource utilization, which contain 7–9 questions each on the readiness survey. We chose these subcomponents based on their high relevance in our previous rapid qualitative analysis that examined *R* = MC^2^ subcomponents in implementing general and CRC screening-related practice changes to improve CRC screening in FQHCs [18].

Table 1 presents organizational readiness subcomponents and definitions. Readiness was measured on a Likert scale from 1 (strongly disagree) to 7 (strongly agree), with an additional response option to denote “don’t know/not applicable’ responses. We obtained a clinic-level score for each subcomponent based on participants’ responses within each clinic. For example, we summed responses to all items related to *priority* from X number of participants at Clinic A and calculated the mean; this is then the priority score for Clinic A.

**Table 1.** Organizational readiness subcomponents and definitions

	Subcomponent	Definition
**Motivation**	Priority	Importance of the innovation compared to other things a clinic does.
	Compatibility	The innovation fits with how a clinic does things.
	Observability	Ability to see that the innovation is leading to visible and desired outcomes.
**Innovation-Specific Capacity**	Inter-organizational Relationships	The necessary relationships between organizations (external to a clinic) that support an innovation.
	Intra-organizational Relationships	The necessary relationships within an organization that support an innovation.
**General Capacity**	Resource Utilization	Ability to acquire, allocate, and use resources.
	Organizational Structure	Method by which works flows through a clinic.

### Potential confounding variables and study size

We controlled for two variables in our analysis using clinic-level data from the clinic contact survey: 1) how long the clinic has been open (ordinal variable: less than one year, 1–3 years, four or more years), and 2) clinic size (operationalized by a continuous variable: number of clinical personnel [e.g., providers] in the clinic). We chose both variables because FQHC clinics vary greatly in *size* and *length of service*, which can affect implementation outcomes [19,20]. Finally, the unit of measurement for this analysis was at the clinic level, with a sample size of 57 clinics, which included individuals nested within each clinic. Given the exploratory nature of this study, we interpreted and reported effect sizes rather than p-values. We used a correlation of 0.30 or higher because it represents a moderate effect size [21].

### Analysis

#### Linear analysis

We computed descriptive statistics for the survey respondents’ characteristics, including age, gender, race, ethnicity, time worked in the current position and at the current FQHC clinic, clinic role, and level of education. We examined clinic characteristics from each participating clinic, including the corresponding state of each FQHC, number of personnel (clinic size), number of medical visits in the previous calendar year, proportion of patients (aged 50 and over) who were up-to-date on CRC screening in the previous calendar year, primary test recommended for CRC screening at the clinic for average-risk individuals, and time clinic has been open.

We then used Pearson correlations to assess bivariate associations between the selected set of organizational readiness variables and implementation levels of EBIs and to note any differences between intervention types. We selected variables in the multivariable models by identifying those with effect sizes greater than or equal to .30. We then computed multivariable linear associations between readiness variables and implementation levels of EBIs while controlling for clinic-level variables (e.g., clinic size). We used an ordinary least squares regression model given the perceived continuous nature of the dependent variable (level of EBI implementation). We conducted additional analyses using a categorical approach to further understand the linear results.

#### Categorical analysis

In our primary statistical analysis, we treated the dependent variable (level of EBI implementation) as continuous. We used one-way analysis of variance (ANOVA) to test group differences in readiness subcomponent mean scores using EBI implementation responses (i.e., no plans to do so, planning to implement, early stages of implementation, inconsistently implemented, and fully implemented). Specifically, we used ANOVA modeling for significant readiness variables from bivariate analyses. Tukey HSD tests assessed post hoc pairwise comparisons. To account for potential confounding variables, we employed an Analysis of Covariance (ANCOVA) model to control for clinic size and time the clinic has been open [22]. All analyses were conducted using the STATA statistical package (version 17; StataCorp LLC, College Station, TX, USA).

## Results

Our sample included 514 respondents from 57 clinics who provided readiness and level of implementation data on surveys. We examined variable distributions and carried out descriptive statistics to characterize the respondents and clinical demographic information. The combined mean of all independent variables (priority, compatibility, observability, inter-organizational relationships, intra-organizational relationships, resource utilization, and organizational structure) was 5.65. The dependent variables of provider reminders and reducing structural barriers averaged 2.87 and showed a non-normal distribution. However, provider assessment, feedback, and patient reminders showed normal distributions with an average score of 3.25.

The most common job types for respondents were medical assistant (*n* = 143), nurse (*n* = 90), and provider (*n* = 84) (Table 2). There were several other reported job types, including administrators and clinic managers. Provider or patient reminders were the most frequently implemented EBIs, as indicated by the number of individual surveys completed. Most clinics had been open for four years or more (*n* = 45) and had an average clinic size (i.e., number of personnel) of 14 individuals and an average of 4 providers per clinic. Clinics had an average of 3,240 medical visits in the past calendar year and most commonly recommended at-home FIT tests for average-risk patients (Table 3).

**Table 2.** Descriptive statistics of study sample survey respondents

	Total (N = 514)	
**Demographics**	n	Percent (%)
Age (Mean, SD)	39.57 (11.83)	
Years in Current Position (Mean, SD)	4.70 (5.40)	
Years Worked in Current Clinic (Mean, SD)	4.29 (5.04)	
**Gender**		
Female	461	89.69
Male	49	9.53
Prefer not to answer	3	0.58
Missing	1	0.19
**Clinic Role**		
Provider (e.g., physician, nurse practitioner)	84	16.34
Nurse	90	17.51
Quality Improvement/Operations/Clinic Manager	34	6.61
Medical Assistant	143	27.82
Other (e.g., patient service representative, referral specialist, case manager, front desk reception)	161	31.32
Missing	2	0.39
**Race and Ethnicity**		
White	311	60.51
Black, African, or African-American	154	29.96
Asian	5	0.97
Native Hawaiian or Other Pacific Islander	2	0.39
Native American, American Indian, or Alaska Native	6	1.17
Hispanic or Latino Ethnicity (of any race)	170	33.07
Other	36	7.00
**Level of Education**		
Some high school	2	0.39
High school graduate or GED	58	11.28
Some college	98	19.07
Associate’s Degree/Diploma/Certificate (e.g., CNA)	184	35.80
College graduate	68	13
Graduate degree or medical degree	104	20

*Note: SD* = Standard deviation; *GED* = General education diploma; *CNA* = Certified nurse aid.

**Table 3.** Clinic characteristics

	Total (N = 57)	
	n	Percent (%)
**FQHC Corresponding State**		
Texas	28	49.12
South Carolina	27	47.40
New York	2	3.51
**Number of Personnel (Clinic Size), (Mean, SD)**	14 (16)	
**Medical Visits in the Previous Calendar Year (Mean, SD)**	3240.13 (4110.10)	
**Proportion of patients (50 and over) up to date on screening in Previous Calendar Year**		
<20%	17	29.82
20–39%	11	19.30
40–59%	17	29.82
60–79%	10	17.54
Missing	2	3.51
**Primary Test Recommended for CRC screening at clinic for average-risk individuals**		
Colonoscopy	28	49.12
At-home FOBT	8	14.04
At-home FIT	46	81.00
At-home FIT-fecal DNA (Cologuard)	33	57.89
**Time Clinic Has Been Open**		
Less than 1 year	3	5.26
1–3 years	5	8.77
4 or more years	45	78.95
Missing	4	7.02

*Note: FQHC* = federally qualified health center; *CRC* = Colorectal cancer; *SD* = Standard deviation.

### Linear analysis results

We used Pearson correlations to determine the association between the selected organizational readiness variables and implementation levels of EBIs. Results showed that organizational structure and resource utilization were inversely associated with implementing patient reminders (*r* = −0.32 and *r* = −0.30, respectively) and were significant at *p* < 0.05 (Table 4). A multivariable linear regression model was used to assess the relation between organizational structure, resource utilization, and the implementation of patient reminders while controlling for other variables (i.e., clinic size and time the clinic has been open). Results demonstrated suggestive evidence that organizational structure was inversely associated with the implementation of patient reminders (β = −0.30, 95% CI = −1.05 – 0.05) while controlling for clinic size and time the clinic has been open (Table 5). Based on unstandardized results, findings imply that a one unit increase in organizational structure is associated with a 0.55 unit decrease in the implementation of patient reminder clinic score while controlling for clinic size and time the clinic has been open.

**Table 4.** Pearson correlations between organizational readiness variables and reported levels of implementation of CRC screening EBIs

Organizational Readiness Variables	Provider Reminders	Patient Reminders	Provider assessment and feedback	Reducing structural barriers
**Priority**	−0.07	−0.08	−0.11	−0.10
**Compatibility**	−0.03	−0.16	−0.19	−0.09
**Observability**	−0.02	−0.19	−0.11	−0.02
**Inter-organizational Relationships**	0.04	−0.19	0.04	0.05
**Intra-organizational Relationships**	−0.00	−0.21	−0.07	0.00
**Organizational Structure**	−0.07	−0.32*	−0.19	−0.11
**Resource Utilization**	−0.08	−0.27*	−0.12	−0.08

*Note: N* = 57 clinics. Boldface text indicates *r* > 0.30 corresponding with a moderate effect size; *CRC* = Colorectal cancer; EBIs = evidence-based interventions. *indicates *p* < .05.

**Table 5.** Regression coefficients for linear regression model for patient reminders

Readiness Variable	Model I *B*	*SE*		95% CI	Model II *B*	β	95% CI
**Organizational structure**	−.56*	0.25		(−1.06 – 0.06)	−.55*	−0.30	(−1.05 – .05)
**Resource utilization**	−0.58	0.30		(−1.18 – 0.02)	−0.57	−0.26	(−1.17 – .03)

*Note: n* = 57. Model II controls for clinic size and time clinic has been open. *p < 0.05; *B* = unstandardized beta coefficient, *SE* = standard error, *β* = Standardized beta coefficient.

### Categorical analysis results

Our linear results indicate that organizational structure and resource utilization were inversely associated with the implementation of patient reminders. Given these unanticipated results, we used ANOVA models to understand what could be driving differences in a non-linear manner. The overall *F* for the one-way ANOVA was not statistically significant for resource utilization scores, *F*(4,47) = 2.20, *p* = .08. ANOVA results revealed significant group differences between patient reminder implementation groups (e.g., fully implemented) and organizational structure scores. The overall *F* for the one-way ANOVA was statistically significant, *F*(4,47) = 3.62, *p* = 0.01 (Table 6). Furthermore, the Tukey HSD post hoc test results revealed a significant difference between the following groups: 1) inconsistently implemented vs. planning to implement, and 2) inconsistently implemented vs. fully implemented.

**Table 6.** Mean organizational structure scores across implementation groups of patient reminders

	ANOVA					ANCOVA*				
*Implementation Groups*	*N*	*Mean*	*SD*	*F (df)*	*η2*	*N*	*Mean*	*SD*	*F (df)*	*η2*
No plans to do so	10	5.78	0.45	3.62(4)**	0.64	10	6.66	0.45	4.39(4)**	0.64
Planning to implement	12	5.74	0.60			12	6.06	0.60		
Early stages of implementation	7	5.80	0.50			7	6.41	0.50		
Inconsistently implemented	13	4.85	1.13			13	4.60	1.13		
Fully implemented	10	5.84	0.63			10	6.13	0.63		

*Note: SD* = standard deviation, *F* = F-statistics, *df* = degrees of freedom, *η* ^*2*^ = eta-squared; *ANCOVA model adjusted for clinic size and time clinic has been open. **p < .01.

Specifically, the inconsistently implemented group had a lower mean value than the comparative groups, with a difference of −0.89 (*p* = .04) and −0.99 (*p* = .01), respectively. The ANCOVA model demonstrated similar results, indicating significant group differences *F*(4, 23) = 4.39, *p* = 0.01 (Table 6). Tukey’s HSD post hoc test results confirmed the mean differences between the two groups: 1) inconsistently implemented vs. planning to implement (−1.45; *p* = .04) and 2) inconsistently implemented vs. fully implemented (−1.53; *p* = .01). Overall, results suggest that organizational structure scores were significantly lower for clinics that reported inconsistently implementing patient reminders than clinics that reported planning to implement or fully implementing.

## Discussion

This study examined the associations between readiness subcomponents and the reported implementation of CRC screening EBIs in FQHCs. The linear regression model revealed an inverse association between organizational structure, resource utilization, and patient reminders. Given our primary statistical results, we conducted an additional analysis to better understand our findings. The ANOVA results were not significant for resource utilization scores but revealed significant group differences between the patient reminder implementation groups and organizational structure scores. In particular, clinics that reported inconsistent implementation of EBIs had lower organizational structure scores than clinics that planned to implement or fully implemented EBIs.

This study identified organizational structure as a critical factor in the implementation of patient reminders. Specifically, we found evidence of an inverse relation between organizational structure and the implementation of patient reminders. This finding indicates that those clinics with higher implementation of patient reminders also reported lower organizational structure scores. This result suggests that aspects of a clinic’s organizational structure may hinder implementing patient reminders.

Organizational structure is a theoretical subcomponent of the general capacity component in organizational readiness, and in our study, refers to how workflow processes occur within a clinic [23]. The patient reminder EBI consists of written (e.g., letter, email) or telephone (e.g., automated voice or text) messages informing patients that they are due for screening [1]. A lower organizational structure score refers to an individual’s negative perception of their clinic’s communication, leadership, and procedures for implementation efforts (see the organizational structure survey items in Table 7). Evidence from previous studies supports the association between organizational structure and the implementation of patient reminders in clinical settings [24,25].

**Table 7.** Organizational structure and resource utilization survey items

Organizational structure	Resource utilization
Our leadership committees (e.g. board, advisory, or steering) actively contribute to clinic goals.	Our clinic can access a wide range of resources for new initiatives.
People in our clinic are able to communicate openly with each other.	Our clinic has a clear financial plan to maintain initiatives.
People in our clinic communicate well with each other.	Our clinic has a clear process to prioritize resources.
Our clinic has effective procedures to resolve internal problems.	Our clinic knows how to use resources effectively.

A qualitative study exploring barriers and facilitators to the use of computerized clinical reminders identified the lack of clinic coordination between nurses and providers as a barrier and the integration of reminders into workflows as a facilitator [26]. A realist review exploring contextual conditions that influence healthcare improvement identified several factors, including organizational capacity and infrastructure, as critical elements to the extent that quality improvement initiatives are planned, implemented, and maintained [27].This finding is substantiated by another systematic review of factors influencing the adoption of communication technologies by healthcare professionals, which also noted organizational environment (including characteristics of the structure of work, time constraints, and workload) as primary barriers to implementation success [28].

Our study showed that organizational structure was the only variable that was associated with the implementation of patient reminders. A likely explanation for the lack of association between organizational structure and other EBIs is that the patient reminder EBI was most frequently implemented (34%). A more representative sample with other EBIs may show associations between organizational structure and other EBIs. The available sample size may be another limiting factor influencing the inability to uncover relationships between other variables and implementation outcomes. It is possible that we did not find any additional positive correlations between the organizational readiness subcomponents and EBI implementation due to missing other confounding variables in our study. One example is measuring the use or ease of using specific electronic health record (EHR) functions, which may help identify barriers to implementing EBIs, especially those integrated into EHRs (e.g., provider reminders). Previous studies have found that variables related to the ease of EHR use influence the implementation of evidence-based care for CRC in community health centers, especially given clinics’ lower capacities and resources [29].

We also found that clinics that reported they “inconsistently implemented” patient reminders had lower mean organizational structure scores. This finding is crucial because EBIs are often inconsistently implemented, which affects their reach and effectiveness [30,31]. We selected an implementation measure to represent several levels of implementation. However, the measure may not have performed well because the “inconsistently implemented” level was at the higher end of the scale. As this level can indicate that a clinic is struggling to implement an EBI across the clinic, it may not belong to the high end of the scale. Therefore, scaling in our study may have resulted in measurement errors [32]. In addition, treating this measure as categorical rather than continuous may be more meaningful given that “inconsistent implementation” is not necessarily a higher level of implementation than “planning to implement.” For example, a clinic could be in the implementation planning phase while avoiding inconsistent implementation with good planning and supportive implementation strategies.

This error refers to inconsistencies across survey items that may not have captured the full extent of implementation at the clinic level. Our study examined the mean EBI implementation score (primary analysis) and explored the relation between implementation categories (additional analysis) and the average readiness scores. It is possible that clinics that reported that they were inconsistently implementing focused on other EBIs or were not as engaged in focusing their workflow efforts on the particular patient reminder EBI. Consequently, there is a need to select effective implementation strategies to enhance inconsistently implemented interventions.

### Strengths and limitations

Our study has some limitations. We recognize the cross-sectional nature of this study does not allow us to make causal inferences between the predictor and outcome variables. In addition, social desirability bias has impacted participants’ responses to be favorable [33]. To address this issue, we prioritized word choice in our survey to ensure the anonymity of survey responses. Another limitation is the measurement of our implementation scale, which we initially thought could represent different implementation levels. However, our results showed that it was limited, suggesting that treating implementation as a categorical variable may be more appropriate. We plan to address this in future survey iterations by using a more comprehensive implementation outcome scale for each EBI. We also acknowledge that the readiness measures used in this study were from a developmental survey; thus, they are not in their final form and will undergo further refinement.

The last limitation of our study is the types of sampling used. We utilized a convenience sampling approach to select clinics to participate in our survey as we approached clinics with which we had some connection to participate in our developmental sample. We also used a purposive sampling approach, sending the survey link to eligible participants through a clinic contact. The potential threat of using these approaches is related to selection bias, in which researchers can make assumptions while choosing specific survey or clinic participants [34,35]. However, we countered this bias in two ways: 1) comparing clinical characteristics by state to represent our sample better, and 2) defining eligibility criteria to require survey participants to have at least some confidence in answering questions about CRCS EBIs. These methods are appropriate for gathering the best possible information from a specific population subset.

This is the first study to examine the magnitude of the associations between organizational readiness subcomponent scores and reported levels of implementation in a clinical context. As such, this study examined the relations between organizational readiness subcomponents and the implementation of four CRC-screening EBIs in FQHCs. In addition, although we had a relatively small sample of clinics, we had a large sample of respondents (*N* = 514). A large sample of respondents allows for improved reliability when using our organizational readiness measure within each clinic.

## Conclusion

It is essential to assess factors influencing organizational readiness and design strategies to accelerate and improve the implementation of CRC screening EBIs in FQHCs. Assessing and building organizational readiness can improve the implementation outcomes of EBIs. Our study examined the relation between organizational readiness and implementation of EBIs to increase CRC screening in FQHCs. We found that the organizational structure subcomponent was associated with implementing patient reminders. We also found that clinics that reported inconsistent implementation had lower organizational structure scores than clinics that planned or fully implemented patient reminders. The results of this study help guide researchers in prioritizing organizational structure (i.e., clinical workflows) and selecting specific implementation strategies to improve this determinant in implementing CRC screening-related EBIs.

Future research should examine these associations using a larger sample size to determine other relations between organizational readiness and implementation outcomes. Findings from this study can also lead to further investigations of how other subcomponents (e.g., inter-organizational relationships) may contribute to the success or inconsistency of CRC screening EBI implementation and longitudinal assessments of how organizational readiness subcomponents can influence patient reminders and other EBIs over time.
